# SLC26 family: a new insight for kidney stone disease

**DOI:** 10.3389/fphys.2023.1118342

**Published:** 2023-05-25

**Authors:** Jialin Li, Sigen Huang, Shengyin Liu, Xinzhi Liao, Sheng Yan, Quanliang Liu

**Affiliations:** ^1^ The First Clinical College, Gannan Medical University, Ganzhou, Jiangxi, China; ^2^ Department of Urology, The First Affiliated Hospital of Gannan Medical University, Ganzhou, China

**Keywords:** SLC26, kidney stone, oxalate, hyperoxaluria, oxalate metabolism

## Abstract

The solute-linked carrier 26 (SLC26) protein family is comprised of multifunctional transporters of substrates that include oxalate, sulphate, and chloride. Disorders of oxalate homeostasis cause hyperoxalemia and hyperoxaluria, leading to urinary calcium oxalate precipitation and urolithogenesis. SLC26 proteins are aberrantly expressed during kidney stone formation, and consequently may present therapeutic targets. SLC26 protein inhibitors are in preclinical development. In this review, we integrate the findings of recent reports with clinical data to highlight the role of SLC26 proteins in oxalate metabolism during urolithogenesis, and discuss limitations of current studies and potential directions for future research.

## Background

Kidney Stone, also known as urolithiasis, is a common disease that has long threatened human health. Its current prevalence of 14.8% (RULE et al., 2020) is increasing worldwide, with a 5-year recurrence rate of up to 50% (URIBARRI et al., 1989). Kidney stones are a direct cause of substantial morbidity and economic burdens, and may be complicated by hydronephrosis, urinary tract infections, and neoplasia.

The etiology of urolithiasis is complex and multifactorial. Urolithogenesis often results from abnormal urine chemistry (HOWLES and THAKKER, 2020). Predispositions include high urinary concentrations of calcium, oxalate, and uric acid (THONGPRAYOON et al., 2020). When the kidney is affected by intrinsic factors (including genetics, nutrition, metabolic abnormalities, urinary tract obstruction, or infection) and extrinsic factors (including natural and socio-economic environmental stressors), the dynamic balance between urine constituents and stone-inhibiting molecules (citrate or magnesium) is disturbed, leading to the precipitation of solutes, cellular debris, bacteria, and other components (MULAY et al., 2017; HAN et al., 2019).

Transporter proteins play important roles in ion homeostasis that are partially understood, and that deserve further study. SLC26 proteins are categorized as solute carriers, the second largest group of human membrane proteins, and affect various functions (MOUNT and ROMERO, 2004). These multifunctional proteins carry various substrates; primarily chloride, bicarbonate, oxalate, sulphate, formate, and other anions; and thereby regulate ion homeostasis (ALPER and SHARMA, 2013).

The SLC26 family of anion transporters plays important roles in the etiology of urolithiasis, mediating the transport of several key molecules essential to stone formation, including oxalate (CRIVELLI et al., 2020; WANG et al., 2021). For example, hyperoxaluria-induced injury of the renal tubular epithelium stimulates expression and secretion of macromolecules such as cadherin, osteoprotegrin, and collagen, thereby promoting the attachment of nascent crystals to the epithelial surface and consequent crystal nucleation, aggregation, and growth (JOSHI et al., 2015; HOWLES and THAKKER, 2020; WITTING et al., 2021). Here, we review the available evidence of the SLC26 protein family’s relationship to the risk of urolithiasis and detail the studies of its function.

## Oxalate and urolithogenesis

The chemical composition of kidney stones is variable. Calcium, oxalate, and phosphate account for 80% of stones, while the remainder are composed of uric acid, struvite, cystine, insoluble drug molecules (such as the HIV protease inhibitor indinavir) and metabolites (KHAN et al., 2017). As one of the most prevalent disorders of ion metabolism in patients with urolithiasis, oxalate dysregulation has long served as a research priority. Sources of oxalate are classified as exogenous (e.g., dietary intake) and endogenous (e.g., hepatic synthesis).

The chronic dietary intake of foods rich in oxalate, especially in its soluble form (e.g., vegetables, nuts, and some cereals), predisposes to hyperoxaluria (MITCHELL et al., 2019) (CHEN et al., 2001). Gastrointestinal diseases and bariatric surgery may increase enteric epithelial permeability (DUFFEY et al., 2010) (TARPLIN et al., 2015), thus causing oxalate hyperabsorption that leads to hyperoxalemia and consequent hyperoxaluria. In addition, gut dysbiosis has been associated with urolithiasis, and particularly with calcium oxalate (CaOx) stones (MILLER et al., 2019). The gut microbiome communicates with enterocytes to perform various biological processes that may impact oxalate metabolism. For example, *Oxalobacter formingenes* stimulates enteric oxalate excretion/secretion and also consumes oxalate as a carbon source and thereby reduces oxaluria (HATCH et al., 2006; ARVANS et al., 2017).

Hepatic synthesis is the primary endogenous source of oxalate. Several autosomal recessive disorders, such as mutations of alanine/glyoxylate amino (AGT) transferase (WOOD et al., 2019), glyoxylate/hydroxypyruvate reductase (HOPPE et al., 2009), and the liver-specific mitochondrial enzyme 4-hydroxy-2-ketoglutaratealdolase-1 (4-hydroxy-2-oxoglutaratealdolase, HOGA1) (MONICO et al., 2011) increase hepatic oxalic acid production and raise the risk of urolithiasis.

## SLC26 family overview

**FIGURE 1:**
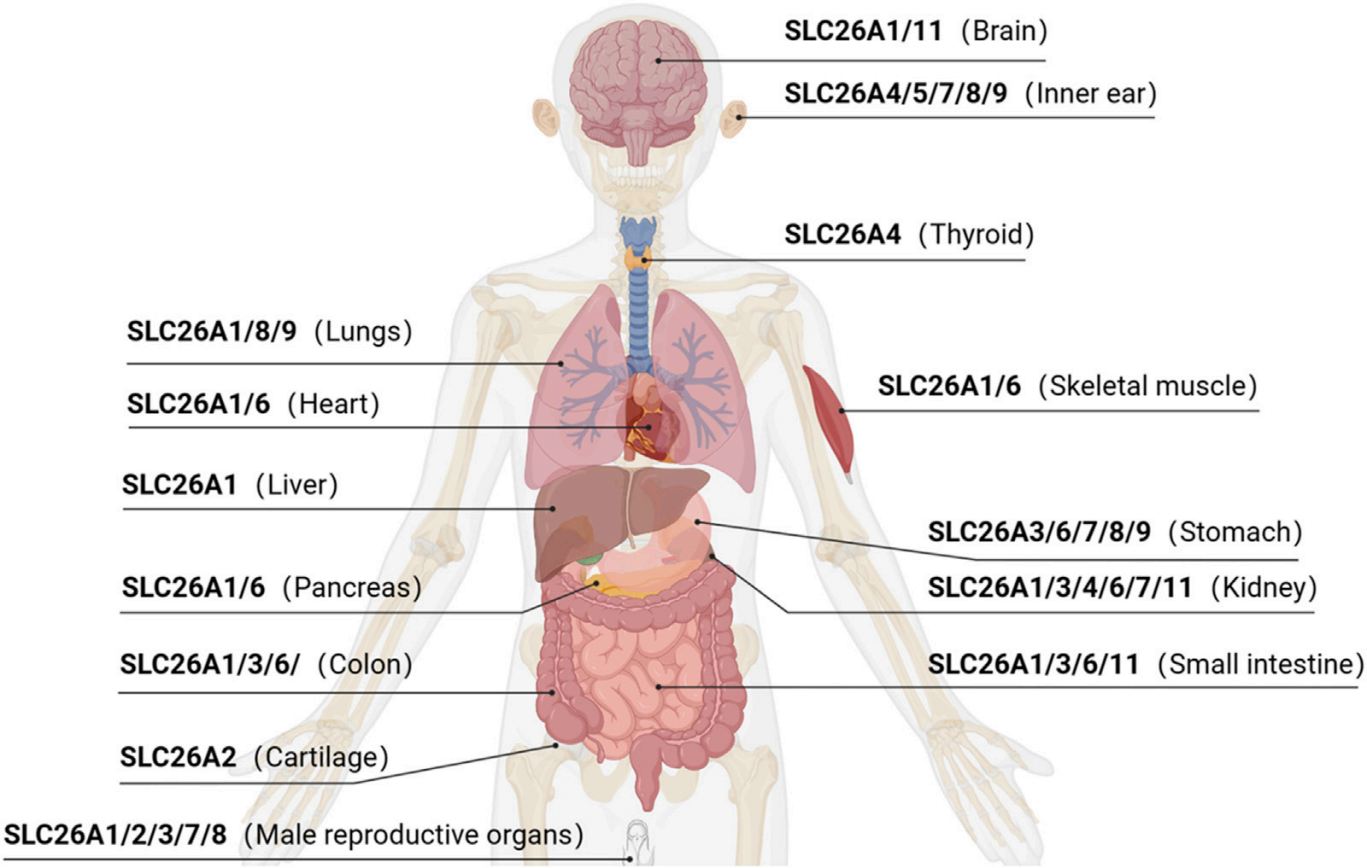
Anatomic distribution of SLC26 protein expressions.

The SLC26 family is an evolutionarily conserved class of transporter proteins encoded by the APC gene superfamily (ALPER and SHARMA, 2013) in taxonomically diverse organisms. Examples include the bacterial protein SLC26/SulP and the plant/yeast-associated protein SLC26/Sultr. Ten isoforms have been identified in humans (among them, SLC26A10 is a transcribed pseudogene), which are expressed across multiple organ systems (Figure 1). SLC26 proteins operate as anion exchangers that transport an anion that is coupled to another anion gradient, but there are a few exceptions. For example, SLC26A5 (prestin) acts as an electrical stress-sensitive motor protein responsible for the electrokinetic behavior of outer hair cells in an anion-dependent manner (BAVI et al., 2021).

**FIGURE 2:**
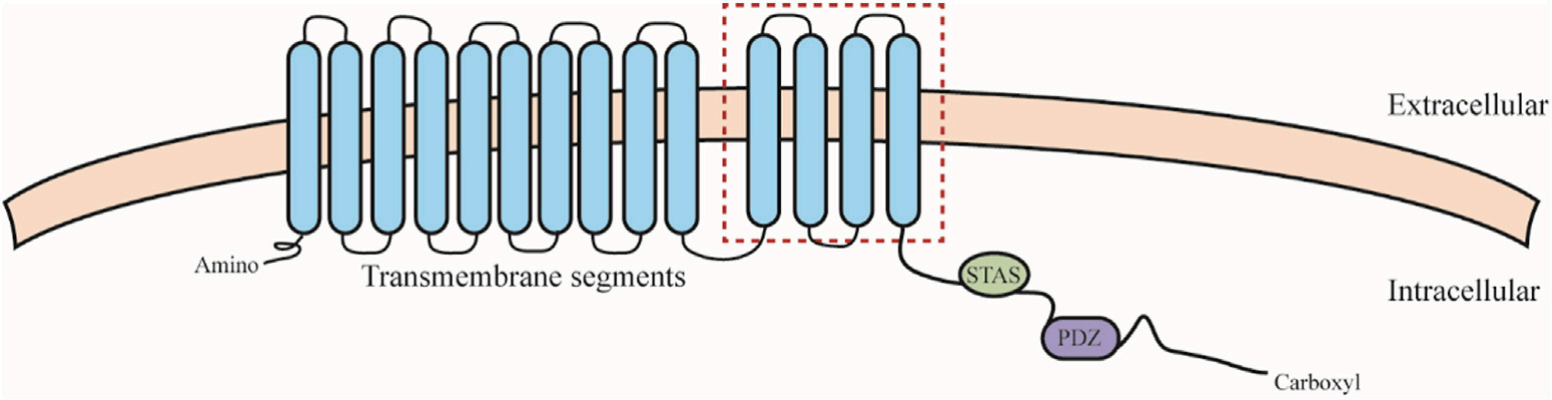
SLC26 protein structure. SLC26 including an N-terminal structural domain, a segment consisting of 10–14 transmembrane segments (the red square indicates differences in hydrophobic span that may exist between members), a C-terminal sulfate transporter and anti-sigma factor antagonist structural domain (STAS), and a PDZ structural domain.

SLC26 family members share similar structures, including an N-terminal structural domain, a segment consisting of 10–14 transmembrane segments, a C-terminal sulfate transporter and anti-sigma factor antagonist structural domain (STAS), and a PDZ structural domain (CHANG et al., 2019) (Figure 2). Remarkably, the highest variability and most functionally significant variations of STAS structural domains are located in a defined region designated as the intervening sequence or variable loop found between the first α-helix and the third β-sheet. This loop interacts with the cystic fibrosis transmembrane conductance regulator (R structural domain in the cystic fibrosis transmembrane regulator) and mediates epithelial chloride and bicarbonate transport (MOUNT and ROMERO, 2004). This function may be cross-regulated by SLC26 and related transporters. SLC26 proteins feature monomeric structures, but often act by forming structural and functional dimers in lipid membranes (TOURé, 2019), which may cause structural-functional alterations of SLC26 *in vivo*.

Pathogenic mutations have been associated with SLC26A1 (CaOx stones) (WHITTAMORE et al., 2019); SLC26A2 (skeletal deformities and abnormal cartilage development) (ZHENG et al., 2019); SLC26A3 (chloride diarrhea) (KUMAR et al., 2021); SLC26A4 (Pendred syndrome, deafness, enlarged vestibular aqueduct syndrome, and thyroid lesions) (YUAN et al., 2020; TREPICCIONE et al., 2021); SLC26A5 (deafness) (BAVI et al., 2021); SLC26A6 (bicarbonate ion metabolism related diseases) (SONG et al., 2012); SLC26A7 (hypothyroidism and gastric neuroendocrine tumors) (CANGUL et al., 2018; ISHII et al., 2019); SLC26A8 (asthenozoospermia) (GAO et al., 2022); and SLC26A9 (cystic fibrosis) (PINTO et al., 2021). No pathogenic SLC26A11 mutants have been reported (Table 1). Accordingly, all mutations in the SLC26 gene family have been characterized through their corresponding models. In summary, the study of SLC26-mediated molecular pathogenesis can elucidate mechanisms of disease and facilitate the discovery of therapeutic targets.

**TABLE1:** SLC26 family member-related diseases.

Protein	Main associated diseases
SLC26A1	calcium oxalate stones, hyperoxalemia
SLC26A2	skeletal deformities and abnormal cartilage development
SLC26A3	chloride diarrhea, alkalosis
SLC26A4	Pendred syndrome, deafness, EVA syndrome, and thyroid lesions
SLC26A5	non-comprehensive deafness
SLC26A6	bicarbonate ion metabolism related diseases
SLC26A7	Hypothyroidism
SLC26A8	sperm weakness disorder
SLC26A9	cystic fibrosis
SLC26A10	N/A
SLC26A11	Dysregulation of chloride homeostasis and neuroactivity

## SLC26 proteins in the kidney

The major SLC26 proteins expressed in human renal tissue are SLC26A1, SLC26A3, and SLC26A6. Molecular, cellular, and *in vivo* studies have confirmed the regulatory role of SLC26 proteins in urolithiasis, as detailed below.

### SLC26A1

SLC26A1, also known as sulfate anion transporter protein-1 (SAT1), is an epithelial transporter that maintains oxalate and sulfate homeostasis. It is expressed primarily in the kidney, liver, and intestine. SLC26A1 exhibits high homology (78% amino acid identity) and similar tissue distribution between humans and mice (LEE et al., 2005; SEIDLER and NIKOLOVSKA, 2019), suggests the usefulness of animal models as preclinical models for studying SLC26 family proteins. A rat model of CaOx nephrolithiasis showed that SLC26A1 was expressed primarily on the basolateral membranes of renal proximal tubular and enteric epithelia, thus affecting oxalate absorption and excretion (GEE et al., 2016). SLC26A1 deletion alters the dynamic balance between oxalate and sulfate, causing hyperoxalemia and consequent hyperoxaluria, renal calcium deposits, and CaOx urolithiasis, and may also predispose to acetaminophen-induced hepatotoxicity (DAWSON et al., 2010). In addition, intense leukocytic infiltration was detected in the perivascular renal cortex of SLC26A1-deficient mice, suggesting the involvement of inflammatory pathways in this process. Meanwhile, sulfate homeostasis, another important factor in lithogenesis, is also disturbed, shown by hyposulfatemia and hypersulfaturia, which together with disturbances in oxalate metabolism increased the risk of stone formation.

The introduction of gene profiling into clinical practice enabled the identification of SLC26A1abnormalities in patients with kidney stones. Twenty-one clinically significant missense mutations of SLC26A1 were identified in patients with nephrolithiasis (ZIYADOV et al., 2021). Detection of SLC26A1 variant expression and comparison with the NCBI database showed that loss of function was associated with decreased protein stability (no significant difference in the number of transcripts). Notably, sequencing of a specimen from a patient with severe renal calcinosis revealed a substitution of a non-conserved amino acid (M132T) in the transmembrane structural domain of SLC26A1 (DAWSON et al., 2013), thus altering the structure of the hydrophobic region of the third segment, suggesting that the loss of SLC26A1 protein function may lead to hyperoxaluria and urolithiasis. The above evidence suggests that understanding the function of SLC26A1 at the genetic level may be a useful strategy.

### SLC26A3

SLC26A3 mediates chloride and bicarbonate exchange and regulates the balance between oxalate secretion and reabsorption. It is expressed primarily in the intestine, kidney, and adrenal glands. Generally, all human intestinal segments engage in a dynamic balance of oxalate absorption and secretion to avoid hyperoxalemia. SLC26A3-KO mice showed a 66% reduction in daily urinary oxalate excretion compared to wild-type mice, indicating the importance of the SLC26A3 transporter in intestinal oxalate uptake. Furthermore, in the same study, SLC26A6 levels were increased 3-fold in the duodenum and jejunum, suggesting a possible complementarity between SLC26A3 and SLC26A6 expressions (FREEL et al., 2013). However, another study showed that SLC26A3 overexpression in the murine intestine did not increase oxaluria and also reduced renal CaOx deposition, suggesting a role in preventing rather than promoting urolithiasis (LIU et al., 2021). These lines of evidence suggests that abnormal SLC26A3 expression, a risk factor for urolithiasis, is related to other mechanisms of urolithogenesis in addition to the regulation of oxalate metabolism.

Over the past decade, researchers have suggested that SLC26A3 may serve as a therapeutic target to treat hyperoxaluria and prevent CaOx urolithiasis. CIL et al. (2022) demonstrated that DRAinh-A270 selectively inhibits SLC26A3-mediated chloride/bicarbonate exchange. Notably, the same study reported that the reduced function of SLC26A3 mutants, which may decrease male fertility, may be caused by a weakened interaction between the specific structural domain STAS and the cystic fibrosis transmembrane conductance-regulated channel, thereby affecting chloride transport. To date, no studies have confirmed the role of SLC26A3 in renal physiology, but its role in the regulation of enteric anion exchange suggests that targeting SLC26A3 may represent a novel strategy to prevent CaOx urolithiasis, as it regulates oxalic acid metabolism in the body to reduce the risk of kidney stones caused by hyperoxaluria. However, further studies are needed to confirm this hypothesis.

### SLC26A6

SLC26A6, also known as PAT-1, is a chloride/oxalate transporter that is highly expressed in the kidney, pancreas, and intestine, and plays an important role in urolithogenesis. MONICO et al. (2008) first identified and characterized a SLC26A6 variant arising from a mutation in the STAS structural domain (D23H/D673N) in hyperoxaluric patients. This change impairs SLC26A6 expression and function, and disrupts the dynamic balance between citrate and oxalate, thereby promoting urolithogenesis (MONICO et al., 2008). This missense mutation is often complicated by hypertension and cystic fibrosis (SHIMSHILASHVILI et al., 2020). These findings have led to a growing interest in the relationship between SLC26A6 and kidney stones. Furthermore, JIANG et al. (2018) showed that renal but not systemic SLC26A6 hyperexpression increased oxalate secretion by renal tubular epithelial cells. *In vitro* experiments showed that elevated oxalate concentrations stimulated the intracellular production of reactive oxygen species and pro-inflammatory factors, leading to further cellular injury and crystal nucleation (JIANG et al., 2018). This finding also corroborates the above-mentioned involvement of the SLC26 family in inflammatory signaling.

Citrate prevents pathological mineralization of CaOx by inhibiting early nucleation, during the early stages of stone formation, there is often a dysregulation of citrate metabolism. Urinary citrate is reabsorbed mainly through NaDC-1, a Na+-dicarboxylic acid cotransporter in the proximal tubule. Increased renal NaDC-1 activity enhances tubular reabsorption of citrate and facilitates CaOx crystallization (JIANG et al., 2018). OHANA et al. (2013) found that the STAS structural domain of SLC26A6 interacted with the first intracellular loop (ICL1), a functional structural domain of NaDC-1, to inhibit NaDC-1 activity, thereby limiting citrate uptake, suggesting that the STAS-ICL1 interaction underlies the dynamic regulation of citrate/oxalate exchange by SCL26A6/NaDC-1, and that feedback between proteins the two balances their expression and function. This interaction is partially mediated by the scaffolding protein IRBIT through stimulation of the succinate receptor SUCNR-1 (KHAMAYSI et al., 2019).

SLC26A6 expression is regulated by a variety of factors, such as inflammation and miRNA. Protein kinases A (PKA) may positively regulate SLC26A6 levels and alter its intrinsic activity by increasing its expressions on the apical membranes of renal and intestinal epithelia. At the initiation of stone formation, PKA activation not only upregulates SLC26A6 expression, but also enhances the transporter activity of SLC26A2 and SLC26A6 (ARVANS et al., 2020). Similarly, in obesity-induced systemic inflammation, high levels of pro-inflammatory factors can significantly reduce SLC26A6 levels, reversing oxalate transport from net secretion to net absorption, leading to hyperoxalemia/uria, thus promoting urolithogenesis (AMIN et al., 2018). Notably, obesity alters the gut microbiome (MARUVADA et al., 2017). Furthermore, the upregulation of SLC26A6 by short-chain fatty acids (acetate, propionate, and butyrate) produced by gut microbes (LIU et al., 2021) reduced renal CaOx crystals in a rat model; this study suggests a close association between enteric microflora and oxalate metabolism, and also suggests a role of short-chain fatty acid food supplements in the prevention of CaOx urolithiasis.

*In vivo* studies have shown that SLC26A6 is modulated by molecules other than inflammatory mediators. Urinary glycine concentrations were significantly lower in patients with hyperoxaluria and were associated with downregulated SLC26A6 and NaDC-1 post-transcriptional levels via miRNA-411-3p linking the 3′ end untranslated regions of both mRNAs, thus decreasing urinary oxalate/citrate ratios and ultimately reducing CAOx crystallization in the rat kidney (JUNG et al., 2018). Moreover, SLC26A6 expression may be endocrine-dependent, as it may be regulated by parathyroid hormone (TSENG et al., 2020). In summary, SLC26A6 regulates oxalate metabolism, and loss of function variants promote the formation of kidney stones.

### Other SLC26 family members associated with renal calculi

Other proteins of the SLC26 family are closely associated with kidney stones. For example, HIRATA et al. (2012) identified the role of SLC26A5 in CaOx urolithiasis by using a *Drosophila* model that may facilitate genetic studies regarding renal tubular ion transport and CaOx crystallization. Another study simulated the human physiology of SLC26A5 in the *Drosophila* stone model, and showed that in addition to transporting oxalate, it also regulates sulphate levels through competitive transport, thereby decreasing urinary calcium salt saturation and reducing CaOx precipitation (LANDRY et al., 2016).

## Discussion

Recent research has elucidated the role of SLC26 proteins in CaOx urolithiasis. These proteins have been increasingly recognized as important transporters that regulate the homeostasis of ions associated with stone development and have become a focus for drug development. SLC26 family proteins are widely expressed in multiple tissues of the gastrointestinal, urinary, skeletal, and reproductive systems. The expressions of SLC26 family members and their physiologic roles vary considerably between species, suggesting different pathogenic mechanisms of SLC26 mutants between humans and animal models (SOLEIMANI, 2013). Abnormalities of urinary ion concentrations, such as hyperoxaluria, are important pathogenic factors (SIENER and Hesse, 2021). The SLC26 family is centrally involved in oxalate and sulfate ion transport in urolithogenesis, and the STAS structural domain is an important site of action.

The study of SLC26 transporter proteins is still incomplete. For example, SLC26A1 is normally expressed in the intestine and kidney and regulates oxalate absorption and secretion; however, a study that demonstrated renal tubular deposition of calcium oxalate in SLC26A1-KO mice could not associate this finding directly to SLC26A1 deficiency (KO et al., 2012). Concomitant urinary pH and osmolarity may be altered through SLC26-mediated transport; however, no studies have been conducted to test this hypothesis. In addition, SLC26 proteins may reduce the risk of hyperoxaluria and stones to a greater degree in female compared to male rats, suggesting sex-based differences in SLC26 family protein expression and activity (ALPER and SHARMA, 2013). Unfortunately, most animal studies of SLC26 have used male mice. On the other hand, the expression patterns of most SLC26 family proteins are still unclear, which may be due to bias caused by poor reproducibility in animal models and low specificity of corresponding antibodies, resulting in divergent results (DAWSON et al., 2010; KO et al., 2012; GEE et al., 2016; WHITTAMORE et al., 2019). However, it is noteworthy that genetic studies of patients and animal models have revealed multiple variants of specific SLC26 proteins, which may serve as drug targets for the prevention of urolithiasis. The development of SLC26 protein-targeting drugs has made some progress, with the identification of small-molecule drug candidates, such as DRAinh-A270, which exhibits protein inhibition *in vitro*. However, potential side effects and clinical utility are still unknown, and are currently being explored. Future work will advance the understanding of SLC26 proteins and the development of therapeutic strategies based on their multiple physiologic roles.
